# Characteristics of women on opioid substitution therapy in primary healthcare in Tshwane (South Africa): a retrospective observational study

**DOI:** 10.3399/BJGPO.2024.0049

**Published:** 2025-01-29

**Authors:** Daniela S Goeieman, Robert Mash, Natasha R Gloeck, Andrew Scheibe

**Affiliations:** 1 Department of Family and Emergency Medicine, Faculty of Medicine and Health Sciences, Division of Family Medicine and Primary Care, Stellenbosch University, Stellenbosch, South Africa; 2 Division of Clinical Associates, Department of Family Medicine and Primary Care (DFMPC), School of Clinical Medicine, Faculty of Health Sciences, University of the Witwatersrand, Johannesburg, South Africa; 3 Health Systems Research Unit, South African Medical Research Council, Cape Town, South Africa; 4 Department of Family Medicine, Faculty of Health Sciences, University of Pretoria, Pretoria, South Africa

**Keywords:** community based primary care, harm reduction, opiate substitution treatment, primary health care

## Abstract

**Background:**

Women who use drugs face specific challenges compared with men such as higher rates of HIV infection, unsafe injecting practices, and intimate partner violence (IPV). However, this population’s access to drug dependence treatment and gender-sensitive interventions remains limited, leading to unmet needs and increased vulnerability.

**Aim:**

To investigate the characteristics of and associations with retention in care among women on opioid substitution therapy (OST) in a community-based primary care setting.

**Design & setting:**

A descriptive observational study within the Community Orientated Substance Use Programme in Tshwane, South Africa.

**Method:**

Data from 199 women (aged >18 years) on OST was extracted from an electronic database and paper-based files. Data were analysed descriptively, and inferential analysis looked for association of variables with retention on OST for ≥6 months.

**Results:**

The majority of participants were unemployed, with 44.3% aged 20–29 years. During the initiation and course of OST, 39.2% of women had an intimate partner of which 37.2% reported IPV, and 19.2% were pregnant. Retention on OST was significantly associated with increasing age at initiation (*P* = 0.047), knowledge of HIV status (*P* = 0.029), an increase in the Alcohol, Smoking and Substance Involvement Screening Test (ASSIST) score (*P* = 0.023), and methadone dose (*P*<0.001). Factors such as race, employment status, health-system level, pregnancy, intimate partner using substances, IPV, route of administering opioids, and having tuberculosis and/or hepatitis C exposure did not show a significant relationship with retention on OST (*P*>0.05).

**Conclusion:**

This study reveals specific vulnerabilities in women receiving OST, emphasising the need for the integration of interventions to address reproductive health, violence mitigation, infectious disease, and polydrug use into care.

## How this fits in

Prior to this research, it was understood that opioid use among women in South Africa was a critical issue, but there were very little data on women as most studies focused predominantly on male participants. This research emphasises the urgent need for gender-sensitive, decentralised harm reduction services to manage opioid use in women. It reveals that women with opioid use disorders face unique challenges and vulnerabilities that require tailored interventions. Clinicians providing community-based addiction care services like opioid substitution therapy should do so in an integrated and patient-centred approach to better support women in overcoming these challenges.

## Introduction

The escalating global prevalence of substance use, which caused 30.9 million healthy life years lost in 2021, underscores the urgency for effective harm reduction interventions.^1^ The high HIV burden among people who inject drugs (PWID) in sub-Saharan Africa,^2–6^ estimated at 21% in South Africa in 2017,^5^ is particularly alarming.

Services often neglect the unique challenges faced by women who use drugs (WWUD).^2,6^ Research data disproportionately focuses on men, contributing to the invisibility of WWUD and their vulnerabilities.^6,7^ Women remain understudied, relevant reports often lack a gender focus, and some interventions do not acknowledge gender differences, or who has access to treatment within the field of substance use.^6^ WWUD are at increased risk of HIV infection from engaging in sex work, challenges in condom negotiation with sexual partners, and exposure to unsafe injecting practices.^8^ Other vulnerabilities experienced by WWUD are mental health issues, intimate partner violence (IPV), having an intimate partner who uses substances, losing child custody, infertility, complications during pregnancy, and stigma. These factors affect women's access to sexual and reproductive health, harm reduction, and other decentralised healthcare services.^6–13^ Across the globe, policymakers, service providers, and WWUD are calling for decentralised gender-sensitive services as this achieves 'optimal access'.^14^

Harm reduction encompasses a range of evidence-based interventions, including opioid substitution therapy (OST).^9,15,16^ These interventions are provided in a non-judgmental environment and reduce adverse health and socioeconomic consequences of substance use.^9^ Using opioid-agonist medications, such as methadone or buprenorphine in OST as maintenance therapy, rather than detoxification, enhances treatment retention, decreases risky behaviours, and effectively addresses associated health issues such as infectious diseases and mental health conditions.^15–19^ Furthermore, OST accompanied by psychosocial support, access to housing, employment, peer support and counselling enhances outcomes.^4^ Where there is gender responsiveness in OST services, women have positive outcomes, particularly those who have parental responsibilities or are pregnant.^4,13,20^

South Africa is one of five African countries that provides community-based OST.^9^ Within South Africa, the Community Orientated Substance Use Programme (COSUP) in Tshwane, Gauteng, exemplifies a commitment to comprehensive harm reduction.^16,21^ However, challenges persist in understanding the utilisation patterns of harm reduction programmes such as COSUP. Recruiting WWUD to participate in studies on OST retention and prevalence of HIV and Hepatitis C is challenging.^22–24^ Furthermore, data from the South African Community Epidemiology Network on Drug Use (SACENDU) suggests an under-representation of women in drug dependence treatment, indicating a gender imbalance.^2^ This raises concerns about the ability of substance use programmes to reach and address the specific vulnerabilities faced by WWUD. This study aimed to evaluate the sociodemographics, substance use patterns, and retention of women using COSUP’s OST services.

## Method

### Study design

This was a descriptive observational study using existing data collected by COSUP.

### Setting

COSUP primarily services urban and peri-urban communities in South Africa’s largest metropolitan city, Tshwane. A city comprising of residential, educational, and industrial areas making it a diverse and representative setting for such a study.

COSUP operated 17 sites located at health and social institutions in the public, non-government, academic, and private sectors. Sites included community health centres, regional hospitals, non-government organisations (NGOs), and homeless shelters. By 2022, COSUP had registered more than 5000 service users. Sites provide harm reduction services, psychosocial support, and safe social spaces through interdisciplinary teams comprising of clinical associates, nurse practitioners, doctors, social workers, peer educators, and community health workers.

The programme screens for risky substance use using the Alcohol, Smoking and Substance Involvement Screening Test (ASSIST; version 3.0), which is a standardised tool that assesses the frequency, quantity, and negative consequences (health, legal, social, and financial) of substance use in the past 3 months.^25,26^ Based on their responses, people are classified into one of the following three risk categories for each drug: lower risk with no intervention (scores from 0–10 for alcohol, 0–3 for all other drugs); moderate risk who receive a brief intervention (scores from 3–27); and higher risk who receive more intensive treatment (scores >27).

### Study population

The study population was all cis and transgender women (aged ≥18 years) with opioid use disorder in Tshwane, initiated on OST by COSUP between December 2016 and January 2022. No sampling was performed; the entire study population was included.

### Data collection

Data were abstracted from electronic databases and paper-based records. The OST database provided information on age, gender, race, location, substance use practices, employment status, infectious diseases (HIV, hepatitis C and tuberculosis [TB] status) on initiation, along with OST type, daily maintenance dose, and reasons for terminating OST. To ensure reliability of the data 15% of the electronic data were cross-checked with the paper-based records. Data on specific risk factors, such as history of gender-based violence, having a sexual partner who uses substances, and history of pregnancy while on OST, were extracted from the paper-based records. Unrecorded data points were marked as missing, and denominators adjusted during analysis.

### Data analysis

The Statistical Package for Social Sciences (SPSS; version 28.0.0.0) was used for data analysis. Categorical data were reported as percentages and frequencies. Numerical data were analysed for mean and standard deviation (SD) or median and interquartile range (IQR), depending on the distribution.

COSUP considers a service user as retained if they remain in OST services for at least 6 months. Initial uninterrupted retention on OST was used as the dependent variable for inferential analysis. The total months on OST, despite interruptions before the 6 month mark, were calculated by summing durations across all initiation episodes. Mean total ASSIST score was calculated by averaging the individual ASSIST scores across multiple drugs for each participant before the initiation of OST. To assess factors associated with retention and nominal categorical variables (age, race, employment status, health facility, route of administering opioids, HIV infection, hepatitis C exposure, and type of agonist), the Kruskal–Wallis test was used. The Mann–Whitney U test was used to assess the association with retention across binary categorical variables (pregnancy while on OST, drug use by intimate partner, IPV, screened for TB, TB infection, and HIV treatment enrolment). To assess the relationship between retention and ASSIST scores, Spearman’s correlation was used.

## Results

### Characteristics on initiation of OST

Table 1 presents a summary of the characteristics of the 199 women at OST initiation. The mean age was 31.5 (SD 7.5) years with 44% aged 20-29 years. Almost all were cisgender (99.5%) and the majority identified as Black women (66.8%). Most were seen in non-governmental facilities (141, 70.9%), run independently or in partnership with the University of Pretoria. During the period of either initiating or being maintained on OST, 19.2% of the participants were pregnant. Few participants had formal employment, with less than 6.0% having the means to generate their own income.

**Table 1.** Characteristics of women on initiation of opioid substitution therapy

Variable	*n* (%)
**Age in years on initiation (*n* = 183**)	
16–19	2 (1.1)
20–29	81 (44.3)
30–39	77 (42.1)
40–49	15 (8.2)
50–59	8 (4.4)
**Race (*n* = 199)**	
Black	133 (66.8)
Coloured^a^	17 (8.5)
White	44 (22.1)
Indian or Asian	5 (2.5)
**Gender (*n* = 199)**	
Cisgender women	198 (99.5)
Transgender women	1 (0.5)
**Pregnant at initiation or while on OST (*n* = 125)**	24 (19.2)
**Employment status (*n* = 184)**	
Unemployed	174 (94.6)
Employed	6 (3.3)
Self-employed	4 (2.2)
**Type of health facility where OST was accessed (*n* = 199)**	
Community venues run by non-governmental organisation	56 (28.1)
Community venues run by University of Pretoria	8 (4.0)
PHC facilities run by non-governmental organisations or University of Pretoria	77 (38.7)
PHC facilities run by local municipality	18 (9.0)
PHC facilities run by provincial government	25 (12.6)
District hospitals	8 (4.0)
Regional hospital	7 (3.5)

^a^Coloured is South African's race classification for individuals of mixed race or ethnicity. OST = opioid substitution therapy. PHC = primary health care.

### Intimate partner substance use and violence

Overall, 78 (39.2%) reported having an intimate partner on initiation and while on OST. Of these partners, 64 (82.1%) used substances and intimate partner violence (IPV) was reported by 29 (37.2%) women.

### Substance use practices

In 55.4% (*n* = 102/184) of women, the most common route of administering opioids was injection, followed by smoking (42.4%, *n* = 78/184), snorting (1.1%, *n* = 2/184), or ingesting over the counter opioids (1.1%, 2/184). A substantial proportion of the women scored high risk for opioids (87.3%), cannabis (22.7%), tobacco (22.1%), and cocaine (16.0%). The mean total ASSIST score was 8.0 (SD 2.3) (Table 2).

**Table 2.** Risk categories for substance use as assessed by the ASSIST 3.0 score (*n* = 181)

Substance	Low or no risk, *n* (%)	Moderate risk, *n* (%)	High risk, *n* (%)
Tobacco products	8 (4.4)	133 (73.5)	40 (22.1)
Alcohol	158 (87.3)	18 (9.9)	5 (2.8)
Cannabis	85 (47.0)	55 (30.4)	41 (22.7)
Cocaine	132 (72.9)	20 (11.0)	29 (16.0)
Amphetamine-type stimulants	165 (91.2)	13 (7.2)	3 (1.7)
Inhalants	179 (98.9)	1 (0.6)	1 (0.6)
Sedatives and sleeping pills	167 (92.3)	11 (6.1)	3 (1.7)
Hallucinogens	180 (99.4)	1 (0.6)	0 (0.0)
Opioids	0 (0.0)	23 (12.7)	158 (87.3)
Other drugs	180 (99.4)	1 (0.6)	0 (0.0)

### Communicable diseases and treatment enrolment

Seventy-four women (37.2%) were living with HIV and 48 (24.1%) had an unknown HIV status. Among those living with HIV, 75.7% (56/74) were receiving antiretroviral treatment. Overall, 145 (72.9%) of all women were screened for TB, and six (4.1%) were diagnosed and started on treatment. Hepatitis C prevalence was difficult to assess with only 36 (18.1%) tested, among whom 18 (50.0%) tested positive.

### OST type, dose, and duration on treatment

Methadone was prescribed in 175 (87.9%) of women, with a median daily dose of 40 mg (IQR 30 mg–50 mg), followed by buprenorphine-naloxone (*n* = 22, 11.1%) with a median daily dose of 8 mg (IQR 4 mg–8 mg) and tramadol (*n* = 2, 1.0%) with a median daily dose of 350 mg (IQR 300 mg–400 mg). The median duration of uninterrupted OST for women was 6.0 months (IQR 1.5–17.7). When including the total number of months on OST, for those that were re-initiated after terminating treatment, the median increased to 7.1 (IQR 1.9–17.9) months.

### Reasons for terminating OST

Women discontinued therapy for different reasons (Table 3). Lost to follow-up (LTFU) with no known reason for discontinuation (43.6%) and returning to substance use (12.9%) were the most common reasons.

**Table 3.** Reasons for termination of opioid substitution therapy (*n* = 140)

Reasons	*n* (%)
LTFU, reason unknown	61 (43.6)
Returned to substance use	18 (12.9)
Self-discharged	15 (10.7)
LTFU, following poor adherence to OST programme	11 (7.9)
Deceased	11 (7.9)
Relocated	10 (7.1)
In-patient rehabilitation	8 (5.7)
Incarcerated	5 (3.6)
Hospitalised	1 (0.7)

LTFU = lost to follow-up. OST = opioid substitution therapy

### Factors associated with retention on OST

Supplementary Table 1 presents factors associated with retention on OST. Age categories were significantly associated with retention on OST (*P* = 0.047) and increasing age was also significantly correlated (*r* = 0.210, *P* = 0.004). Unknown HIV status was associated with significantly worse retention compared with those who knew their status (*P* = 0.029). An increasing ASSIST score (*r* = 0.171, *P* = 0.023) and dose of methadone (*r* = 0.339, *P*<0.001) were also positively correlated with retention. Other factors were not associated with retention on OST (*P*>0.05). There were insufficient data to assess associations for gender or tramadol with retention on OST.

## Discussion

### Summary

The majority of WWUD were young, unemployed, cisgender, and accessed OST at NGO facilities. More than one in three women experienced IPV, and 19.2% were pregnant while on OST. In addition to opioid dependence, participants had significant risks associated with tobacco, cannabis, and cocaine. Approximately one-quarter had unknown HIV status, although HIV prevalence was notable. LTFU was the main reason for discontinuation. Methadone was the primary agonist, and retention improved with higher age at initiation, knowledge of HIV status, higher methadone dose, and higher mean total ASSIST score. The counterintuitive link between a higher mean total ASSIST score and improved retention may be clarified by considering that this mean score reflects the cumulative severity of polydrug use assessed before OST initiation, suggesting a potential link between the complexity of substance dependence and a need for longer duration of treatment engagement. The other factors measured were not associated with retention in the non-adjusted analysis.

### Strengths and limitations

This study contributes to the available literature on WWUD in South Africa and offers an accurate description of the women on OST in COSUP. Data accuracy was verified using medical records, particularly for time on OST (retention). The variables that could be analysed were also limited to the data that COSUP routinely collected. The survey design and limited variables might have impacted inferential statistical power. However, there were few borderline *P* values, suggesting that a lack of power was not a major issue. The retrospective cross-sectional design hinders establishing causal relationships, warranting caution in interpreting effectiveness of OST for women with an opioid disorder. Factors influencing or confounding these associations may exist, and regression modelling could have provided deeper insights.

### Comparison with existing literature

Aligned with broader local and global studies,^2,27–30^ the results affirm that women susceptible to substance use disorders, typically aged 18–35 years, unemployed, and using injected substances, seek treatment at non-governmental facilities. The utilisation of non-governmental services, aligns with international standards emphasising the role of NGOs, location, staffing, and provider attitudes.^15^

The study delves into the intersectionality of factors impacting WWUD, including substance use practices and related harms such as IPV and communicable disease and their sexual and reproductive health needs.^15^ Pregnancy motivates treatment enrolment with some women being characterised as 'future-orientated', and motherhood seen as a chance for a 'drug-free future'.^31^ However, pregnant women on OST face challenges in managing substance use, compounded by a ‘stigmatised identity’ and their own guilt or fears about potential harm to their infant.^32,33^ This not only serves as a barrier to accessing services, but also points to a gap in the literature. Existing studies tend to prioritise the effects of substance use on the foetus and infant, neglecting the holistic experiences of pregnant women accessing harm reduction services.^32–34^

Despite the absence of specific associations with individual drugs, the comprehensive evaluation through the mean ASSIST scores suggests that the use of other substances has an effect on OST retention. Such high-risk substance use warrants enrolment in harm reduction services as per guidelines.^35,36^ OST is effective in reducing risky opioid, stimulant, and benzodiazepine use post-initiation, although no reduction has been observed in cannabis or daily alcohol use.^37^

The prevalence of IPV was higher than the national prevalence of 13.1% reported in 2017. The age range, urban dwelling, socioeconomic status, and association between IPV and substance use have been found in other studies.^27,38–40^ In mapping out harm reduction services across the globe, the Women and Harm Reduction International Network found that women in Africa reported that harm reduction services were limited and male-dominated, and that they were stigmatised or discriminated against and subjected to IPV.^41^

Historically, COSUP primarily addressed unregulated substance use and not infectious diseases.^42^ This may explain the low screening, testing and treatment enrolment rates for HIV, TB, and hepatitis C virus (HCV). With HCV being largely asymptomatic, there is a need to implement screening as part of every encounter with PWUD.^43,44^ The HIV prevalence rate of 37.2% for women who use drugs is very close to the 36% recorded in another study for the Tshwane area. It is, however, higher than the prevalence of 26% recorded across three metropolitan areas in South Africa.^18^ In the syndemic context of HIV and opioid use, awareness of HIV status was positively associated with retention. The risk of HIV, akin to pregnancy, may have heightened health consciousness, leading them to take steps towards enrolling in OST. This may reflect the importance of comprehensive care in harm reduction that addresses individual needs and promotes coordinated care for those living with HIV.^45^

While the study primarily examined women, it does confirm that higher doses of methadone facilitate retention on OST.^23^ Further exploration is warranted to understand the impact of accumulated months on OST for women with treatment interruptions. Considering both attempt frequency and continuous use, rather than solely 6-month retention, prompts a nuanced investigation into the dynamics of treatment interruptions and re-initiations on overall OST outcomes for women. In assessing the clinical effectiveness of OST for maintenance, methadone (60 mg–100 mg daily) provides better retention than buprenorphine (8 mg–16 mg daily) and both medications are safe and recommended during pregnancy.^46,47^ In lower to middle-income countries, methadone is more commonly consumed than buprenorphine, potentially owing to the higher cost of agonist medications. In South Africa, for example, the cost of a 100 ml bottle of methadone 10 mg/ml solution ranges from US$22–32 (approximately 17–25 GBP; equivalent to US$1–2 [approximately 0.78–1.55 GBP] per standard minimum dose of 60 mg), while for buprenorphine 2 mg tablets, it ranges from US$1.44–2.26 (approximately 1.12–1.76 GBP; equivalent to US$6–9 [approximately 4.66–7 GBP] per 8 mg dose).^48^

### Implications for research and practice

The findings signal the urgent need for comprehensive interventions targeting both the biological and psychosocial aspects of opioid disorders in WWUD, with a particular focus on pregnant women. Treatment programmes should prioritise sexual reproductive health and rights of WWUD. Enhanced engagement and follow-up strategies are essential to reduce LTFU rates. Additionally addressing IPV, risks of communicable diseases, and polydrug use is crucial based on findings; suggesting further research explores these complex interactions.

To achieve better treatment outcomes and enhance overall wellbeing, collaborative efforts and further research, particularly qualitative studies, are required to address stigma, assess strategies for economic empowerment, ensure comprehensive healthcare services, and develop targeted approaches to address the intricate dynamics between substance use, IPV, and communicable diseases.

## Data

The data relied on in this article are not publicly available. Access would require permission from the University of Pretoria's Community Orientated Substance Use Programme's Research Unit; contact via 4th author (AS).

## References

[1] United Nations Office on Drugs and Crime (2021). World Drug Report 2021..

[2] Dada S, Harker Burnhams N, Erasmus J, Lucas Charles Parry W (2020). South African community epidemiology network on drug use. SACENDU Report. https://www.samrc.ac.za/sites/default/files/attachments/2022-07/SACENDUFullReportPhase48.pdf.

[3] Degenhardt L, Peacock A, Colledge S (2017). Global prevalence of injecting drug use and sociodemographic characteristics and prevalence of HIV, HBV, and HCV in people who inject drugs: a multistage systematic review. Lancet Glob Health.

[4] The Global Fund (2022). Technical brief: Harm reduction for people who use drugs — priorities for investment and increased impact in HIV programming. Allocation period 2023–2025.

[5] South African National AIDS Council (2019). Midterm Review of the National Strategic Plan for HIV, TB and STIs 2017–2022.

[6] International AIDS Society (2019). Women who inject drugs: overlooked, yet visible.

[7] National Institute on Drug Abuse (2021). Substance use in women research report: summary.

[8] Azim T, Bontell I, Strathdee SA (2015). Women, drugs and HIV. Int J Drug Policy.

[9] Harm Reduction International (2020). Global state of harm reduction 2020.

[10] Bardwell G, Austin T, Maher L, Boyd J (2021). Hoots and harm reduction: a qualitative study identifying gaps in overdose prevention among women who smoke drugs. Harm Reduct J.

[11] Valencia J, Alvaro-Meca A, Troya J (2020). Gender-based vulnerability in women who inject drugs in a harm reduction setting. PLoS One.

[12] Medina-Perucha L, Scott J, Chapman S (2019). A qualitative study on intersectional stigma and sexual health among women on opioid substitution treatment in england: implications for research, policy and practice. Soc Sci Med.

[13] Jacobs AA, Cangiano M (2018). Medication-assisted treatment considerations for women with opiate addiction disorders. Prim Care.

[14] McCarthy JJ, Jones HE, Terplan M (2021). Changing outdated methadone regulations that harm pregnant patients. J Addict Med.

[15] World Health Organization, United Nations Office on Drugs and Crime (2020). International standards for the treatment of drug use disorders: revised edition incorporating results of field-testing (Geneva: WHO, UNODC).

[16] Guise A, Seguin M, Mburu G (2017). Integrated opioid substitution therapy and HIV care: a qualitative systematic review and synthesis of client and provider experiences. AIDS Care.

[17] Nielsen S, Tse WC, Larance B (2022). Opioid agonist treatment for people who are dependent on pharmaceutical opioids. Cochrane Database Syst Rev.

[18] Scheibe A, Young K, Moses L (2019). Understanding hepatitis B, hepatitis C and HIV among people who inject drugs in South Africa: findings from a three-city cross-sectional survey. Harm Reduct J.

[19] Csete J, Kamarulzaman A, Kazatchkine M (2016). Public health and international drug policy. Lancet.

[20] Schiff DM, Nielsen TC, Hoeppner BB (2021). Methadone and buprenorphine discontinuation among postpartum women with opioid use disorder. Am J Obstet Gynecol.

[21] Scheibe A, Shelly S, Hugo J (2020). Harm reduction in practice — The Community Oriented Substance Use Programme in Tshwane. Afr J Prim Health Care Fam Med.

[22] Lefoka MH, Netangaheni TR (2021). A plea of those who are affected most by HIV: the utterances by women who inject Nyaope residing in the city of Tshwane municipality, Gauteng. Afr J Prim Health Care Fam Med.

[23] Gloeck NR, Harris BN, Webb EM, Scheibe A (2020). Factors predicting 6-month retention among people with opioid use disorders accessing outpatient methadone maintenance therapy in Tshwane, South Africa. S Afr Med J.

[24] Scheibe A, Makapela D, Brown B (2016). HIV prevalence and risk among people who inject drugs in five south arican cities. Int J Drug Policy.

[25] World Health Organization (2010). The Alcohol, Smoking and Substance Involvement Screening Test (ASSIST): manual for use in primary care. https://www.who.int/publications/i/item/978924159938-2.

[26] Bhoora U, Gloeck NR, Scheibe A (2022). Managing acute opioid withdrawal with tramadol during COVID-19 lockdown in a peri-urban setting. Afr J Prim Health Care Fam Med.

[27] Gibbs A, Jewkes R, Willan S, Washington L (2018). Associations between poverty, mental health and substance use, gender power, and intimate partner violence amongst young (18–30) women and men in urban informal settlements in South Africa: a cross-sectional study and structural equation model. PLoS One.

[28] Karlsson N, Kåberg M, Berglund T (2021). A prospective cohort study of risk behaviours, retention and loss to follow-up over 5 years among women and men in a needle exchange program in Stockholm, Sweden. Int J Drug Policy.

[29] Mwangi C, Karanja S, Gachohi J (2019). Depression, injecting drug use, and risky sexual behavior syndemic among women who inject drugs in Kenya: a cross-sectional survey. Harm Reduct J.

[30] Lawrinson P, Ali R, Buavirat A (2008). Key findings from the WHO collaborative study on substitution therapy for opioid dependence and HIV/AIDS. Addiction.

[31] Knight KR (2015). Addicted. Pregnant. Poor.

[32] Tsuda-McCaie F, Kotera Y (2022). A qualitative meta-synthesis of pregnant women’s experiences of accessing and receiving treatment for opioid use disorder. Drug Alcohol Rev.

[33] Mburu G, Ayon S, Tsai AC (2018). “Who has ever loved a drug addict? It’s a lie. they think a ‘teja’ is as bad person”: multiple stigmas faced by women who inject drugs in coastal Kenya. Harm Reduct J.

[34] Bullinger LR, Wang V, Feder KA (2022). Effects of opioid treatment programs on child well-being. Ann Am Acad Pol Soc Sci.

[35] South African Addiction Medicine Society (2015). South African Guidelines for the Management of Opioid Use Disorders.

[36] Humeniuk R, Henry-Edwards S, Ali R (2010). The Alcohol, Smoking and Substance Involvement Screening Test (ASSIST): manual for use in primary care.

[37] Degenhardt L, Clark B, Macpherson G (2023). Buprenorphine versus methadone for the treatment of opioid dependence: a systematic review and meta-analysis of randomised and observational studies. Lancet Psychiatry.

[38] Lambdin BH, Bruce RD, Chang O (2013). Identifying programmatic gaps: inequities in harm reduction service utilization among male and female drug users in Dar es Salaam, Tanzania. PLoS ONE.

[39] Marsh JC, Amaro H, Kong Y (2021). Gender disparities in access and retention in outpatient methadone treatment for opioid use disorder in low-income urban communities. J Subst Abuse Treat.

[40] Mthembu J, Mabaso M, Reis S (2021). Prevalence and factors associated with intimate partner violence among the adolescent girls and young women in South Africa: findings the 2017 population based cross-sectional survey. BMC Public Health.

[41] Women and Harm Reduction International Network (WHRIN) (2021). Global mapping of harm reduction services for women who use drugs.

[42] Scheibe A, Shelly S, Hugo J, Mohale M (2020). Harm reduction in practice — The Community Oriented Substance Use Programme in Tshwane. Afr J Prim Health Care Fam Med.

[43] Heffernan A, Cooke GS, Nayagam S (2019). Scaling up prevention and treatment towards the elimination of hepatitis C: a global mathematical model. Lancet.

[44] Scheibe A, Sibeko G, Shelly S (2020). Southern African HIV clinicians society guidelines for harm reduction. S Afr J HIV Med.

[45] Sebastian MP, Dasgupta A, Saraswati LR (2017). Service utilization and cost of implementing a comprehensive HIV prevention and care program among people who inject drugs in Delhi, India. Harm Reduct J.

[46] Connock M, Juarez-Garcia A, Jowett S (2007). Methadone and buprenorphine for the management of opioid dependence: a systematic review and economic evaluation. Health Technol Assess.

[47] Kessler SH, Schwarz ES, Liss DB (2022). Methadone vs. buprenorphine for in-hospital initiation: which is better for outpatient care retention in patients with opioid use disorder?. J Med Toxicol.

[48] Clinton Health Access Initiative (CHAI) (2023). HCV market intelligence report. https://chai19.wpenginepowered.com/wp-content/uploads/2023/12/CHAI-HCV-Market-Intelligence-Report-2023.pdf.

